# A meta‐analysis comparing efficacy and safety between proton beam therapy versus carbon ion radiotherapy

**DOI:** 10.1002/cam4.7023

**Published:** 2024-02-23

**Authors:** Jeong Yun Jang, Kangpyo Kim, Miao‐Fen Chen, Tetsuo Akimoto, Michael Lian Chek Wang, Min‐Ji Kim, Kyunga Kim, Tae Hoon Lee, Gyu Sang Yoo, Hee Chul Park

**Affiliations:** ^1^ Department of Radiation Oncology, Samsung Medical Center Sungkyunkwan University School of Medicine Seoul Republic of Korea; ^2^ Department of Radiation Oncology Chang Gung Memorial Hospital Taoyuan Taiwan; ^3^ Division of Radiation Oncology and Particle Therapy National Cancer Center Hospital East Chiba Japan; ^4^ Department of Radiation Oncology National Cancer Center Hospital East Chiba Japan; ^5^ Division of Radiation Oncology National Cancer Centre Singapore Singapore Singapore; ^6^ Biomedical Statistics Center, Research Institute for Future Medicine Samsung Medical Center Seoul Republic of Korea; ^7^ Department of Radiation Oncology Chungbuk National University Hospital Cheongju Republic of Korea

**Keywords:** carbon ion radiotherapy, meta‐analysis, oncologic outcome, particle beam radiotherapy, proton beam therapy, toxicity

## Abstract

**Background:**

This study aimed to compare the outcomes of proton beam therapy (PBT) and carbon ion radiotherapy (CIRT) by a systematic review and meta‐analysis of the existing clinical evidence.

**Methods:**

A systematic literature search was performed to identify studies comparing the clinical outcomes of PBT and CIRT. The included studies were required to report oncological outcomes (local control [LC], progression‐free survival [PFS], or overall survival [OS]) or adverse events.

**Results:**

Eighteen articles comprising 1857 patients (947 treated with PBT and 910 treated with CIRT) were included in the analysis. The pooled analysis conducted for the overall population yielded average hazard ratios of 0.690 (95% confidence interval (CI), 0.493–0.967, *p =* 0.031) for LC, 0.952 (95% CI, 0.604–1.500, *p =* 0.590) for PFS, and 1.183 (0.872–1.607, *p =* 0.281) for OS with reference to CIRT. The subgroup analyses included patients treated in the head and neck, areas other than the head and neck, and patients with chordomas and chondrosarcomas. These analyses revealed no significant differences in most outcomes, except for LC in the subgroup of patients treated in areas other than the head and neck. Adverse event rates were comparable in both groups, with an odds ratio (OR) of 1.097 (95% CI, 0.744–1.616, *p =* 0.641). Meta‐regression analysis for possible heterogeneity did not demonstrate a significant association between treatment outcomes and the ratio of biologically effective doses between modalities.

**Conclusion:**

This study highlighted the comparability of PBT and CIRT in terms of oncological outcomes and adverse events.

## 1 INTRODUCTION

Particle beam radiotherapy (PBRT), a form of radiation therapy (RT), can deliver high radiation doses to tumors and exert antitumor effects. Notably, PBRT distinguishes itself through a distinctive depth‐distribution characteristic known as the Bragg peak.^1^ This characteristic allows high doses to be delivered to the tumor while minimizing exposure to nearby normal tissues. Moreover, carbon ion RT (CIRT) and proton beam therapy (PBT) have been increasingly utilized, and over 250,000 patients have undergone PBRT until 2019. The availability of PBRT has been expanding with over 100 facilities offering this special treatment.^2^

Although some physical differences exist between proton and carbon‐ion beams with respect to the widths of the penumbra and fragmentation tail, these particle beams are generally considered to exhibit similar physical profiles.^3^ However, because of its higher relative biological effectiveness (RBE) and linear energy transfer compared to proton beams, CIRT is expected to have superior biological effectiveness.^4^ Nonetheless, studies on PBRT are mostly single‐arm studies, which may be undervalued when comparing the oncological outcomes of the two treatment modalities. While a few prospective randomized controlled trials (RCTs) and meta‐analyses have compared the treatment outcomes and toxicities between the two modalities of PBRT, the available evidence still needs to be provided. Moreover, no meta‐analyses have focused exclusively on literatures comparing the two treatment arms, PBT versus CIRT.

Therefore, we aimed to systematically review and generalize the published clinical evidences, specifically comparing the treatment outcomes and toxicities between PBT and CIRT.

## 2 MATERIALS AND METHODS

### 2.1 Search strategy and selection criteria

Systematic literature searches were conducted to identify all available articles on the clinical outcomes of PBRT, with the last date of the search until the 1st June 2023. The first search query identified studies using PBT or CIRT, and the second query included all types of tumors that were known candidates for PBRT. The Cochrane Library, PubMed, and EMBASE electronic databases were used, and the keywords to conduct literature searches were (“particle” OR “heavy ion” OR “carbon ion” OR “carbon radiation” OR “radiation therapy technique” OR “Cion” OR “CIRT” OR “c ion rt”) AND (“cancer” OR “tumor” OR “neoplasm” OR “carcinoma” OR “chordoma” OR “sarcoma”). Additional manual searches of references were also performed. Studies were included if they were written in English and met the Population, Intervention, Comparison, Outcome, and Study (PICOS) criteria defined as follows: Population (P) was defined as human subjects, Intervention (I) with all types of PBRT, Comparison (C) with comparison between PBT and CIRT, Outcomes (O) with any oncologic outcomes including local control (LC), progression‐free survival (PFS), overall survival (OS), and any adverse events (AE), and Study (S) was defined as only RCTs or case–control studies. This study was registered in PROSPERO (Protocol No: CRD42023450927).

### 2.2 Data extraction

Four investigators extracted the literature's general characteristics (Jang, JY, Kim, K, Lee, TH, and Yoo, GS). The recorded data included the name of the first author, year of publication, study design, treatment type, sample size, dose per fraction, number of fractions, type of disease, site of the treated area, total dose, pre‐RT treatments, and the study population (age and sex). The sample size and number of events related to treatment outcomes and the occurrence of AE were recorded according to the treatment arm. To compensate for the heterogeneity of dose per fraction and the number of fractions, we used a biologically effective dose (BED) with an alpha–beta ratio of 3 for toxicity evaluation and 10 for oncologic outcome evaluation. The 3‐year and 5‐year LC, PFS, and OS rates were extracted from each study. Concerning AE, we extracted data on the most frequently reported toxicities common to both treatment groups, ensuring consistency in the analysis.

### 2.3 Quality assessment

We performed a quality assessment of all the studies included in the analysis. Four individual radiation oncologists used the star‐based Newcastle–Ottawa Scale. Each item in the assessment could receive a maximum of one star, except for comparability, which could receive one or two stars. The quality of the literature was converted to the Agency for Healthcare Research and Quality standards and was categorized as good, fair, or poor quality based on the following criteria: 3 or 4 stars in the selection domain AND 1 or 2 stars in the comparability domain AND 2 or 3 stars in the outcome/exposure domain for good quality; 2 stars in the selection domain AND 1 or 2 stars in the comparability domain AND 2 or 3 stars in the outcome/exposure domain for fair quality; 0 or 1 star in the selection domain OR 0 stars in the comparability domain OR 0 or 1 stars in the outcome/exposure domain for poor quality.^5^

### 2.4 Statistical analysis

The Biomedical Statistics Center of our institution conducted the statistical analyses. Statistical analysis was executed using R 4.2.3 (Vienna, Austria; http://www.R‐project.org/), packages “metafor” and “meta”. To determine the estimated effect of particle beams on treatment outcomes and toxicities, we extracted or calculated the log hazard ratio (HR) and standard error (SE) for LC, PFS, and OS using Parmar's method, and the log odds ratio (OR) and SE for AE.^6^, ^7^ All HRs and ORs were calculated using CIRT as a reference and the ratio of PBT to CIRT. A random‐effects model was consistently used for the overall population, whereas a fixed‐effects model was employed for subgroup analysis. Heterogeneity was measured using the Higgins and Green I^2^ test.^8^ I^2^ ranged between 0% (no heterogeneity) and 100% (maximal heterogeneity), and the heterogeneity of the study was considered substantial (*p <* 0.1) by Cochran's *Q*‐test and I^2^ >50%. We also evaluated the potential publication bias using Egger's regression test and funnel plots.^9^ For the meta‐regression analysis, we used inverse‐weighted mixed‐effects regression models to evaluate the effect of radiation dose on the occurrence of oncological outcomes and AE.^10^ Statistical significance was set at *p <* 0.05 as statistically significant.

## 3 RESULTS

### 3.1 Selected articles and characteristics

**FIGURE 1:**
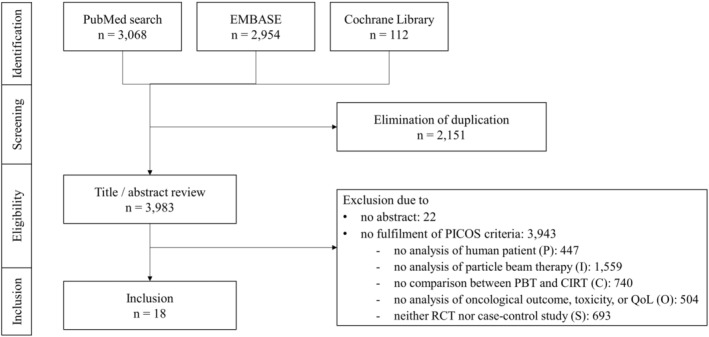
PRISMA flow chart of literature search and selection. PICOS, Population, Intervention, Comparison, Outcome, and Study design.

Figure 1 presents the literature search results, and 3,983 articles were initially identified from three electronic databases. A total of 1,857 patients from 18 selected articles, with 947 receiving PBT and 910 receiving CIRT, were included in the comparative analysis based on the PICOS criteria.^11^, ^12^, ^13^, ^14^, ^15^, ^16^, ^17^, ^18^, ^19^, ^20^, ^21^, ^22^, ^23^, ^24^, ^25^, ^26^, ^27^, ^28^ The characteristics of the included articles are shown in Table 1. Except for two, all were retrospective studies. Among the treated sites, there were 10 articles on the head and neck (including the paranasal sinus, nasal cavity, and skull base), four on the lung, two on the liver, and two on the pelvis. Concerning the type of tumor, the analysis included five articles on skull base tumors, comprising three articles on chordomas and two on chondrosarcomas. In addition, there were three articles on non‐small cell lung cancer; two on adenoid cystic carcinoma of the head and neck; two on hepatocellular carcinoma; and one each on mucosal melanoma, squamous cell carcinoma, any malignancy of the head and neck, oligometastatic disease of the lung, sacral chordoma, and prostate cancer. The number of studies reporting each outcome was 12 for LC, nine for PFS, 13 for OS, and 11 for AE.

**TABLE 1:** Characteristics of the included studies.

First author (year)	Study design	Treated site/histology	Patient recruitment	Country	Affiliation	Source	Quality^a^	Reported outcomes			
								LC	PFS	OS	AE
Iwata (2010)^14^	R	Lung/NSCLC	2003–2007	Japan	Nagoya City Univ.	*Cancer*	Fair	Y	Y	Y	Y
Komatsu (2011)^11^	R	Liver/HCC	2001–2009	Japan	Kobe Univ.	*Cancer*	Fair	‐	Y	Y	Y
Fujii (2013)^26^	R	Lung/NSCLC	2003–2009	Japan	HIBMC	*Radiother Oncol*	Fair	Y	Y	Y	Y
Takagi (2014)^27^	R	H&N/ACC	2002–2012	Japan	HIBMC	*Radiother Oncol*	Fair	Y	Y	Y	‐
Sulaiman (2014)^18^	R	Lung/oligometastatic disease	2003–2011	Japan	HIBMC	*Radiat Oncol*	Poor	Y	‐	‐	‐
Demizu (2014)^21^	R	H&N/mucosal melanoma	2003–2011	Japan	HIBMC	*Strahlenther Onkol*	Fair	Y	Y	Y	Y
Mima (2014)^22^	R	Sacrum/chordoma	2005–2011	Japan	HIBMC	*BR J Radiol*	Poor	Y	Y	Y	‐
Fujii (2015)^20^	R	Lung/NSCLC	2003–2012	Japan	HIBMC	*Acta Oncol*	Poor	Y	Y	Y	‐
Toyomasu (2018)^15^	R	PNS, NC/SCC	2001–2012	Japan	HIBMC	*Int J Radiat Oncol* *Biol Phys*	Fair	‐	‐	‐	Y
Takagi (2018)^28^	R	Skull base/chordoma	2003–2014	Japan	Teishinkai Hospital	*Radiat Oncol*	Poor	Y	Y	Y	Y
Mattke (2018)^13^	R	Skull base/chondrosarcoma	2009–2014	Germany	Univ. of Heidelberg	*Cancer*	Fair	Y	‐	Y	Y
Iannalfi (2020)^24^	P	Skull base/chordoma	2011–2018	Italy	CNAO	*Neuro Oncol*	Good	Y	‐	Y	‐
Hu (2020a)^16^	R	PNS, NC/ACC	2015–2019	China	SPHIC	*Front Oncol*	Poor	‐	‐	‐	Y
Hu (2020b)^17^	R	PNS, NC/an malignancy	2015–2019	China	SPHIC	*Cancer Med*	Poor	‐	‐	‐	Y
Riva (2021)^19^	R	Skull base/chondrosarcoma	2011–2020	Italy	CNAO	*Cancers (Basel)*	Poor	‐	‐	‐	Y
Eichkorn (2022)^25^	P	Prostate	2012–2013	Germany	Heidelberg Univ. Hospital	*Radiother Oncol*	Good	‐	Y	Y	‐
Omiya (2023)^12^	R	Liver/HCC	2000–2015	Japan	Kobe Univ.	*J Am Coll Surg*	Poor	Y	‐	Y	‐
Mattke (2023)^23^	R	Skull base/chordoma	2009–2014	Germany	Heidelberg Univ. Hospital	*Strahlenther Onkol*	Good	Y	‐	Y	Y

^a^ Newcastle‐Ottawa quality assessment scale was used.

Abbreviations: ACC, adenoid cystic carcinoma; AE, adverse event; CNAO, National Center for Oncological Hadrontherapy; H&N, head and neck; HCC, hepatocellular carcinoma; HIBMC, Hyogo Ion Beam Medical Center; LC, local control; NC, nasal cavity; NSCLC, non‐small cell lung cancer; OS, overall survival; P, prospective study; PFS, progression‐free survival; PNS, paranasal sinus; R, retrospective study; SCC, squamous cell carcinoma; SPHIC, Shanghai Proton and Heavy Ion Center.

### 3.2 Oncologic outcomes in the overall population: LC, PFS, and OS

Fourteen studies presented oncologic outcomes with clinical characteristics and 3‐year and 5‐year rates provided in Table 2. The median patient age ranged from 40.5 to 78 years for PBT and from 39 to 75 years for CIRT. The median total RT dose for PBT was 66.0 GyRBE (range, 58.0–76.0), with a median BED of 88.8 GyRBE_10_ (range, 81.3–104.9). For CIRT, the median total dose was 65.0 GyRBE (range, 52.8–70.4), with a median BED of 101.4 GyRBE_10_ (range, 78.0–123.6). For 3‐year LC, PFS, and OS rates, PBT demonstrated ranges of 52.0%–100.0%, 15.0%–98.0%, and 43.7%–100.0%, respectively; the corresponding rates of CIRTs were 61.3%–95.0%, 53.0%–87.0%, and 60.5%–98.0%.

**TABLE 2:** Clinical characteristics and treatment outcomes of the included studies.

First author (year)	Treated site	Type of PBRT	No. of patients	Median age, years	Percentage of males, %	RBE	Dose, GyRBE		LC		PFS		OS	
							Total	BED^a^	3 year	5 year	3 year	5 year	3 year	5 year
Iwata (2010)^14^	Lung	P	37	78	86.0	1.1	60.0	96.0	80.0	N/A	48.0	N/A	70.0	N/A
		C	23	75	60.9	2.37	52.8	122.5	86.0	N/A	74.0	N/A	87.0	N/A
Komatsu (2011)^11^	Liver	P	242	N/A	75.2	1.1	76.0	104.9	90.2	90.2	N/A	N/A	58.7	38.0
		C	101	N/A	72.3	2.0–3.7^d^	52.8	123.6	93.0	93.0	N/A	N/A	60.6	36.3
Fujii (2013)^26^	Lung	P	70	45	71.4	N/A	60.0	96.0	81.0	81.0	44.0	30.0	72.0	62.4
		C	41	39	63.4	N/A	52.8	122.5	78.0	66.6	53.0	36.0	76.0	43.5
Takagi (2014)^27^	H&N	P	40	61.5	35.0	1.1	65.0	81.3	82.0	75.8	60.0	35.0	81.0	54.0
		C	40	58.5	32.5	3.0	65.0	81.3	87.0	77.7	74.0	45.0	88.0	88.0
Sulaiman (2014)^18^	Lung	P	18	66^b^	61.7^b^	1.1	58.0	101.5	52.0	N/A	N/A	N/A	N/A	N/A
		C	23			2.0–3.7^d^	64.0	115.2	61.3	N/A	N/A	N/A	N/A	N/A
Demizu (2014)^21^	H&N	P	33	70	57.6	1.1	65.0	81.3	71.0	71.0	15.0	10.1	43.7	29.0
		C	29	72	58.6	2.0–3.7^d^	65.0	81.3	N/A	N/A	N/A	N/A	N/A	N/A
Mima (2014)^22^	Sacrum	P	7	70^b^	65.2^b^	1.1	70.4	85.9	100.0	N/A	N/A	N/A	100.0	N/A
		C	16			2.0–3.7^d^	70.4	101.4	91.6	40.6	N/A	N/A	78.9	67.7
Fujii (2015)^20^	Lung	P	98^c^	76^b^	67.3^b^	N/A	60.0	99.6	N/A	N/A	N/A	N/A	N/A	N/A
		C	70^c^			N/A	66.0	109.6	N/A	N/A	N/A	N/A	N/A	N/A
Takagi (2018)^28^	Skull base	P	11	N/A	N/A	1.1	N/A	N/A	N/A	80.0	N/A	72.0	N/A	73.0
		C	13	N/A	N/A	2.0–3.7^d^	N/A	N/A	N/A	92.0	N/A	92.0	N/A	100.0
Mattke (2018)^13^	Skull base	P	22	40.5	36.4	N/A	70.0	84.0	100.0	100.0	N/A	N/A	100.0	100.0
		C	79	46	40.5	N/A	60.0	78.0	95.0	91.0	N/A	N/A	95.0	93.0
Iannalfi (2020)^24^	Skull base	P	65	53	61.5	N/A^e^	74.0	88.8	89.0	84.0	98.0	96.0	93.0	83.0
		C	70	58	60.0	N/A	70.4	101.4	77.0	71.0	87.0	84.0	90.0	82.0
Eichkorn (2022)^25^	Prostate	P	46	69	100.0	1.1	66.0	87.8	N/A	N/A	92.0	85.0	98.0	98.0
		C	45	67	100.0	2.4–3.0^f^	66.0	87.8	N/A	N/A	66.0	50.0	91.0	91.0
Omiya (2023)^12^	Liver	P	145	N/A	N/A	N/A	68.2	83.2	91.0	N/A	N/A	N/A	68.0	54.0
		C	125	N/A	N/A	N/A	63.0	85.1	91.0	N/A	N/A	N/A	74.0	54.0
Mattke (2023)^23^	Skull base	P	36	50	61.1	1.1	74.0	88.8	79.8	60.7	N/A	N/A	91.7	91.7
		C	111	51	56.8	3.0–5.0	66.0	85.8	80.4	64.5	N/A	N/A	91.2	83.3

^a^ BED (GyRBE_10_) was calculated by applying α/β ratios of 10, with calculations rounded to the first decimal place.

^b^ Reporting on the entire population without specifying individual numbers for protons and carbons.

^c^ The number of lesions, rather than the number of patients, is provided.

^d^ RBE values of carbon ion radiotherapy were determined as 2.0–3.7, depending on the depth of the spread‐out Bragg peaks.

^e^ Authors did not provide RBE values but only described the model used.

^f^ The RBE values were calculated using local effect model I.

Abbreviations: BED, biologically effective dose; C, carbon ion radiotherapy; H&N, head and neck; LC, local control; N/A, not available; OS, overall survival; P, proton beam therapy; PBRT, particle beam radiotherapy; PFS, progression‐free survival; RBE, relative biological effectiveness.

**FIGURE 2:**
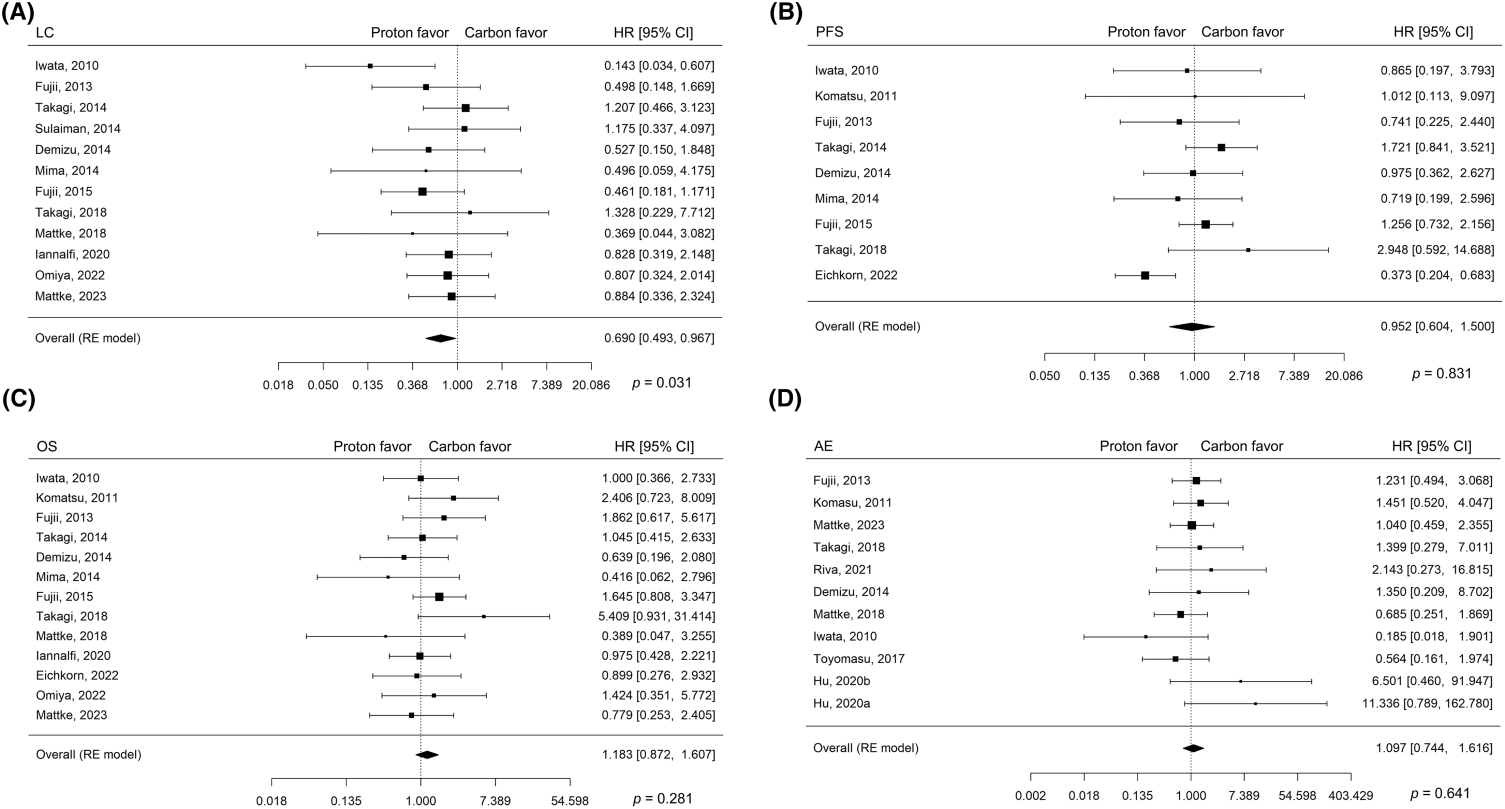
Forest plots with random effect model of pooled analyses regarding (A) local control, (B) progression‐free survival, (C) overall survival, and (D) adverse events. AE, adverse event; CI, confidence interval; HR, hazard ratio; LC, local control; OR, odds ratio; OS, overall survival; PFS, progression‐free survival; RE, random effect.

A pooled analysis was conducted, and the results are presented in Figure 2. The heterogeneity test results are presented in Table 3. Moderate heterogeneity, with an I^2^ of 47.5% (*p =* 0.057), was observed only among the studies on PFS, while the remaining studies showed no heterogeneity. Furthermore, Egger's regression test indicated no publication bias, with funnel plots showing *p*‐values > 0.5 for all outcomes (Table s1). The pooled HR for LC is estimated to be 0.690 (95% confidence interval (CI), 0.493–0.967; *p =* 0.031), indicating a significant difference and favoring PBT. For PFS and OS, the estimated HRs were 0.952 (95% CI, 0.604–1.500; *p =* 0.831) and 1.183 (95% CI, 0.872–1.607; *p =* 0.281), respectively, indicating no significant difference.

**TABLE 3:** Studies included in the pooled analysis for each outcome and analysis for heterogeneity.

Outcome	No. of included study	No. of patients (PBT/CIRT)	Heterogeneity	
			*p*‐value	I^2^ (%)
Local control	12	1222 (582/640)	0.601	0.0
Progression‐free survival	9	962 (584/378)	0.057	47.5
Overall survival	13	1615 (852/763)	0.605	0.0
Adverse event, any grade	11	1049 (528/521)	0.462	0.0
Adverse event ≥ Grade 3	3	134 (76/58)	0.724	0.0

*Note*: I^2^ ≥ 50% suggests high heterogeneity across studies.

### 3.3 Oncologic outcomes in the subgroup population: LC, PFS, and OS

The number of studies used in the subgroup analysis and the heterogeneity test results are shown in Table S2. In the subgroup analysis of patients treated in the head and neck region, the HRs for LC, PFS, and OS were 0.861 (95% CI, 0.536–1.383; *p =* 0.536), 1.542 (95% CI, 0.893–2.661; *p =* 0.120), and 0.965 (95% CI, 0.608–1.531; *p =* 0.880), respectively, using fixed effects model, indicating no significant difference (Figure S1). In the pooled analysis of patients treated in areas other than the head and neck, only PFS showed moderate heterogeneity, with an I^2^ of 44.7%. However, considering the limited number of studies, a fixed‐effects model was employed, yielding HR estimates of 0.551 (95% CI, 0.341–0.890; *p =* 0.015), 0.738 (95% CI, 0.427–1.277; *p =* 0.120), and 1.389 (95% CI, 0.923–2.090; *p =* 0.880) for each outcome (Figure S2). PBT favored LC but showed no significant difference in PFS and OS. Another subgroup analysis was conducted on articles on patients with chordomas and chondrosarcomas. PFS analysis was not conducted because of the limited number of articles available. The HR for LC and OS were 0.809 (95% CI, 0.451–1.449; *p =* 0.476) and 0.956 (95% CI, 0.541–1.689; *p =* 0.877), respectively, demonstrating no significant difference (Figure S3).

### 3.4 Adverse events

A total of 11 studies provided data on AEs, with three reporting the OR for AE ≥ Grade 3. The treatment characteristics and results are presented in Table 4. Heterogeneity tests showed an I^2^ value of 0% for all outcomes. In the pooled analysis of the overall population, the OR for any AE was 1.097 (95% CI, 0.744–1.616; *p =* 0.641) (Figure 2D). Subgroup analyses according to the treatment site and pathology also revealed no significant differences in the occurrence of any AEs between PBT and CIRT (Figure S4). Furthermore, no significant differences were observed in the occurrence of Grade ≥ 3 AEs in the overall population (Figure S5).

**TABLE 4:** Treatment‐related complications in the included studies.

First author (year)	Treated site	Total RT dose, GyRBE (BED^a^)		Adverse event	Percentage of patients with AE		Comments on adverse events
		PBT	CIRT		PBT	CIRT	
Iwata (2010)^14^	Lung	60.0 (180.0)	52.8 (285.1)	Pneumonitis, ≥ Grade 3	2.7	13.0	‐
Komatsu (2011)^11^	Liver	76.0 (172.3)	52.8 (288.6)	Dermatitis, any grade	7.0	5.0	‐
Fujii (2013)^26^	Lung	60.0 (180.0)	52.8 (285.1)	Rib fracture, any grade	25.7	22.0	‐
Demizu (2014)^21^	H&N	65.0 (119.2)	65.0 (119.2)	Any late toxicity, ≥ Grade 3	12.1	6.9	‐
Toyomasu (2018)^15^	PNS, NC	67.6 (126.2)	65.0 (119.2)	Optic nerve disorder, ≥ Grade 3	5.3	19.0	Similar AE between PBT and CIRT No toxicity ≥ Grade 3 (*p* = 0.368)
Takagi (2018)^28^	Skull base	N/A	N/A	Any late toxicity, ≥ Grade 2	54.6	46.2	‐
Mattke (2018)^13^	Skull base	70.0 (116.7)	60.0 (120.0)	Hearing problem, any grade	33.0	40.0	Similar AE between PBT and CIRT No toxicity ≥ Grade 3
Hu (2020a)^16^	PNS, NC	56.0 (93.3)	63.0 (126.0)	Vision impairment, ≥ Grade 3	0.0	1.4	Higher rates of late toxicity with CIRT (patients with re‐irradiation)
Hu (2020b)^17^	PNS, NC	56.0 (93.3)	63.0 (126.0)	Xerostomia, ≥ Grade 3	0.0	11.7	‐
Riva (2021)^19^	Skull base	70.0 (116.7)	70.4 (173.7)	Any late toxicity, ≥ Grade 3	6.3	12.5	‐
Mattke (2023)^23^	Skull base	74.0 (123.3)	66.0 (132.0)	Temporal lobe reaction, any grade	31.0	29.8	Similar AE between PBT and CIRT No toxicity ≥ Grade 4

^a^ BED (GyRBE_3_) was calculated by applying α/β ratios of 3, with calculations rounded to the first decimal place.

Abbreviations: AE, adverse event; BED, biologically effective dose; CIRT, carbon ion radiotherapy; H&N, head and neck; N/A, not available; NC, nasal cavity; PBT, proton beam therapy; PNS, paranasal sinus.

### 3.5 Meta‐regression with BED ratio

A meta‐regression analysis was conducted using the BED ratio to explore the factors that may explain the possible heterogeneity in the HR of oncologic outcomes. However, no significant association was found between the HR of each outcome and the BED ratio (Figure S6). Furthermore, permutation tests were conducted to address the limitation of the small sample size, yielding consistent findings that reinforced the validity of the observed trends (Table S3).

## 4 DISCUSSION

To our knowledge, this is the first meta‐analysis that compares PBT and CIRT exclusively using comparative articles. Despite the difficulty in making direct comparisons owing to the diverse endpoints reported in each study, we observed a degree of comparability in oncologic outcomes and risk of toxicities between the two modalities.

It is widely recognized that although PBT and CIRT share the common advantages inherent to particle beams, they also exhibit distinct properties. Heavy ions exhibit reduced longitudinal and lateral scattering compared to protons, resulting in a smaller dose halo and a narrow penumbra.^29^ Furthermore, a carbon‐ion beam with RBE ranging from 1.5 to 3.4, which is greater than that of a proton beam, is expected to be more effective in eradicating cancer cells with hypoxia and radioresistance.^30^ Given these characteristics, it was expected that CIRT would yield superior oncologic outcomes and reduced toxicity compared with PBT. However, evidences confirming the superiority of CIRT are rare, and this may have been resulted from several reasons. Publishing comparative studies regarding PBT versus CIRT is challenging in the real world because of several factors such as patient preference, insurance coverage, and the limited availability of heavy‐ion centers offering both modalities, resulting in potential bias and limitation in the chance for study conduction. Therefore, the quality of the existing comparative studies is low, and the relevant meta‐analyses included mostly single‐arm studies.^31^, ^32^, ^33^ Furthermore, most studies combined data on photon, proton, and carbon therapies, predominantly emphasizing comparisons between PBRT and photon treatment. The present study is of noteworthy importance as it is the first meta‐analysis on this topic, focusing solely on comparative studies and confirming comparable outcomes between the two modalities. Moreover, the significance of our research was enhanced by incorporating a meta‐regression analysis that aimed to evaluate the effect of radiation dose on outcomes.

Our results indicated a modestly better LC with PBT in the overall population. However, this result requires cautious interpretation because of the potential contribution of the study by Iwata et al., in which the follow‐up duration was at most 35.5 months, and the number of events was only 15.^14^ Therefore, due to the limited quality of this study, it is imperative to interpret these results with caution. Another noteworthy point is that there were no differences in outcomes based on tumor pathology or irradiation site. As sarcomas, including chondrosarcoma and chordoma, are known to be radioresistant compared to other histologies, the potential superiority of CIRT over PBT has often been expected. ^34^, ^35^ Furthermore, CIRT can potentially be more favorable in treating tumors located at the head and neck area because of its distinct physical properties, providing a narrow irradiating volume compared with PBT.^36^

However, the present study showed no significant differences in the oncological outcomes and risk of toxicities in either the sarcoma or head and neck subgroups. Nevertheless, drawing the conclusion that CIRT is not more beneficial than PBT might be premature because several limitations still need to be addressed in its real‐world application. Because of the difficulties in comparing PBT and CIRT in the real world, the number and quality of included studies are small and low, respectively.^37^ Especially most of the included studies were retrospectively conducted, and 44.4% of them showed poor quality based on Newcastle‐Ottawa scale. In fact, most centers that perform CIRT use only fixed‐beam gantries, which restrict the optimization of irradiation angles, thereby limiting the quality of dosimetry.^38^ Furthermore, because the optimal dose prescription and biological model for CIRT have not yet been standardized among institutions, the CIRT protocols among the studies may be diverse.^39^ In particular, the inherent variability of RBE with carbon ions is a major challenge in unifying clinical protocols for CIRT among institutions.^40^, ^41^ Therefore, considering these limitations is crucial when interpreting the findings and drawing conclusions regarding their effectiveness. In the future, the successful integration of modern technologies, such as gantry rotation, along with the establishment and optimization of biological models may offer promising potential for the utilization of carbon ions, particularly in radioresistant histology. Furthermore, as RCTs comparing PBT and CIRT are ongoing, these studies may provide valuable insights into the comparative effectiveness and potential advantages of each treatment modality (NCT01182753, NCT01182779, NCT01165671, NCT01641185, NCT01811394).

Our study had several limitations. To begin with, the restricted number of articles available for analysis stemmed from our stringent inclusion criteria, which focused exclusively on comparative studies. We did not include single‐arm studies to mitigate the potential for an increased risk of bias, and as a result, our analysis was based on a relatively small number of studies.^42^, ^43^ While we made efforts to conduct distinct analyses for various cancer types and organs, we ultimately had to opt for a pooled analysis due to the limited availability of eligible studies. We expect that as high‐quality comparative research continues to emerge, performing more robust meta‐analyses will become increasingly feasible in the future. Moreover, conducting comparative research requires access to both CIRT and PBT within the same institution, which restricted our study to a limited number of centers and possibly introduced potential selection bias. Second, while the majority of the included studies focused on head and neck cancer, followed by lung cancer, prostate cancer is the most frequently treated malignancy using both PBT and CIRT in real world.^44^, ^45^ This discrepancy between publication and utilization in real world is worth noting, and readers should be cautious in their interpretations, considering potential bias. Third, the lack of detailed information on clinical factors such as stage or prior treatment history posed challenges during our analysis. Lastly, the absence of a consensus on the standardized RBE for CIRT has a limitation, as different studies have employed varying RBE values or models. Despite these limitations, our greatest strength lies in our exclusive focus on comparative studies, excluding case reports and series.

## 5 CONCLUSION

PBT and CIRT demonstrated comparable oncological outcomes and toxicities. Nonetheless, the current body of evidence remains equivocal, emphasizing the need for further research to optimize treatment strategies.

## AUTHOR CONTRIBUTIONS

**Jeong Yun Jang:** Data curation (lead); writing – original draft (lead); writing – review and editing (lead). **Kangpyo Kim:** Data curation (lead); investigation (equal); validation (equal); writing – original draft (lead); writing – review and editing (lead). **Miao‐Fen Chen:** Validation (equal); writing – review and editing (equal). **Tetsuo Akimoto:** Validation (equal); writing – review and editing (equal). **Michael Lian Chek Wang:** Validation (equal); writing – review and editing (equal). **Min‐Ji Kim:** Formal analysis (lead); methodology (equal); software (lead); visualization (lead); writing – original draft (supporting); writing – review and editing (supporting). **Kyunga Kim:** Formal analysis (equal); investigation (equal); methodology (equal). **Tae Hoon Lee:** Data curation (equal); methodology (equal); writing – review and editing (equal). **Hee Chul Park:** Conceptualization (lead); supervision (lead); writing – review and editing (lead). **Gyu Sang Yoo:** Conceptualization (lead); data curation (lead); formal analysis (supporting); investigation (lead); methodology (equal); supervision (lead); writing – original draft (equal); writing – review and editing (equal).

## FUNDING INFORMATION

This research did not receive any specific grant from funding agencies in the public, commercial, or not‐for‐profit sectors.

## CONFLICT OF INTEREST STATEMENT

The authors declare that they have no conflicts of interest.

## ETHICS STATEMENT

As a meta‐analysis, ethics approval is not required. No separate IRB review was necessary and not obtained; instead, this study was registered with PROSPERO (Protocol No: CRD42023450927).

## PATIENT CONSENT STATEMENT

Individual patient consent is not required since this involves a meta‐analysis of articles.

## PRECIS

This meta‐analysis of 18 comparative studies reveals that proton beam therapy offers an advantage in terms of local control, while progression‐free and overall survivals remain comparable between particle beam therapies. The study finds that both proton and carbon ion beam therapies exhibit similar risk profiles for adverse events, providing valuable insights into treatment decision‐making for cancer patients.

## Supporting information


Figure S1.
Figure S2.
Figure s3.
Figure s4.
Figure s5.
Figure s6.

Table s1.
Table s2.
Table s3.

## Data Availability

Data supporting the findings of this study are available upon request from the corresponding author.
