# Supplementary material for: A meta‐analysis comparing efficacy and safety between proton beam therapy versus carbon ion radiotherapy
Source: Cancer Med. 2024 Feb 23;13(3):e7023. doi: 10.1002/cam4.7023 (PMC10891363; doi:10.1002/cam4.7023)
Supplement: Supplementary file 1 — Figure S1. Figure S2. Figure s3. Figure s4. Figure s5. Figure s6. [file CAM4-13-e7023-s002.docx]

**Supplementary Figure S1** Forest plots with fixed effect model of pooled analyses involving patients treated with head and neck tumors regarding local control, progression-free survival, and overall survival. LC, Local control; PFS, Progression-free survival; OS, Overall survival; HR, Hazard ratio; CI, Confidence interval; FE, Fixed effect.


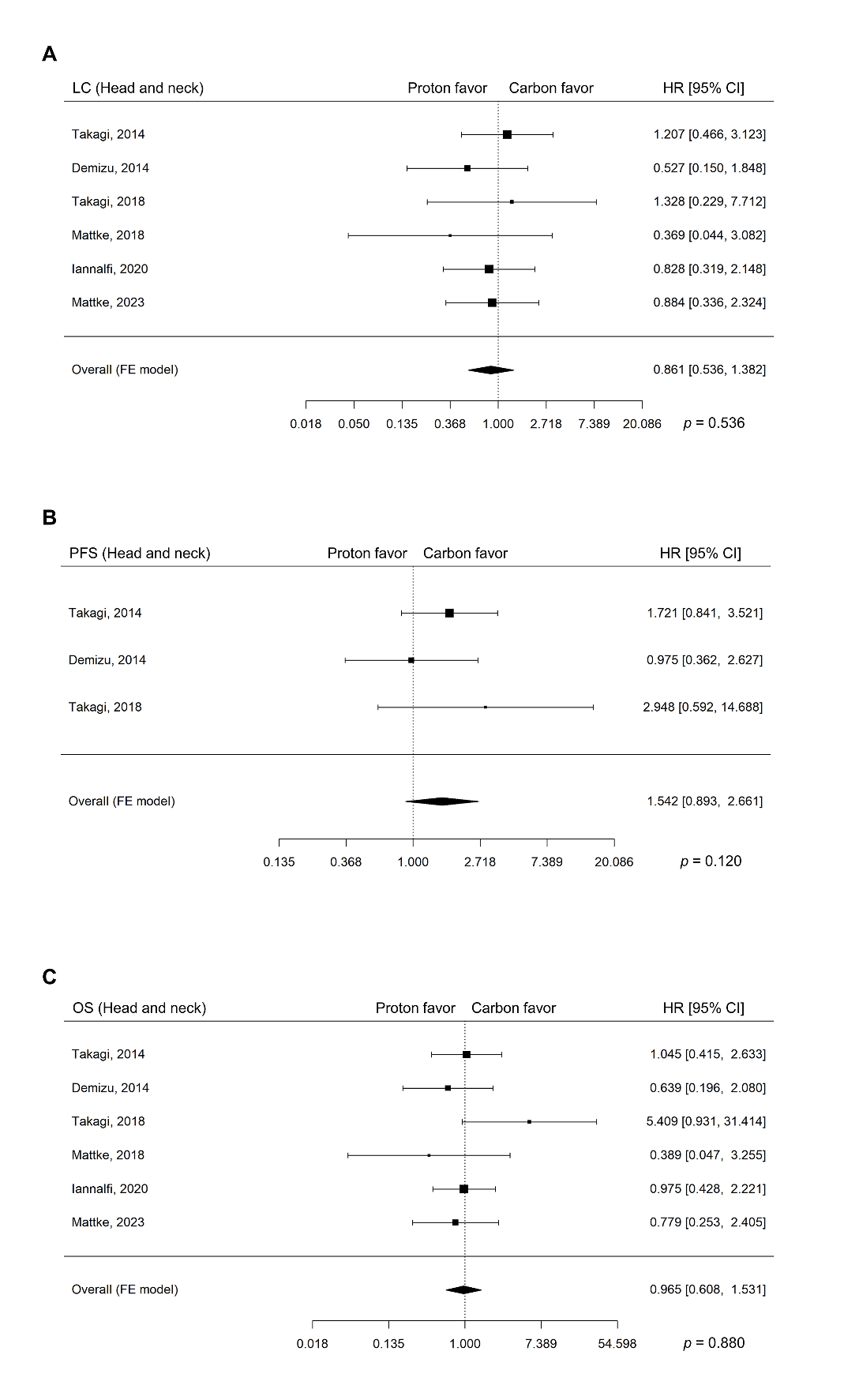


**Supplementary Figure S2** Forest plots with fixed effect model of pooled analyses involving patients treated in areas other than the head and neck regarding local control, progression-free survival, and overall survival. LC, Local control; PFS, Progression-free survival; OS, Overall survival; HR, Hazard ratio; CI, Confidence interval; FE, Fixed effect.

**
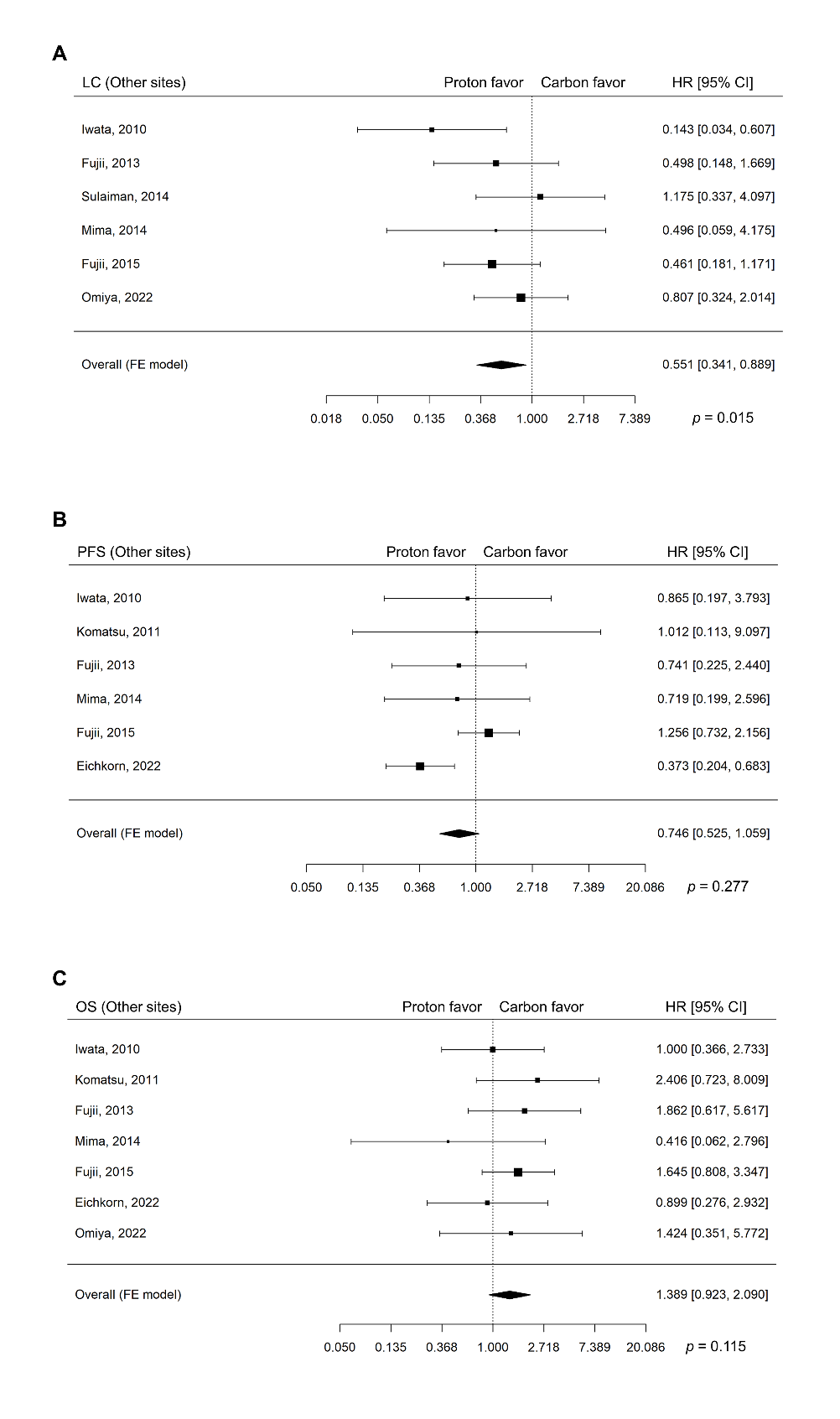
**

**Supplementary Figure S3** Forest plots with fixed effect model of pooled analyses involving patients treated with chordoma or chondrosarcoma regarding local control and overall survival. LC, Local control; OS, Overall survival; HR, Hazard ratio; CI, Confidence interval; FE, Fixed effect.

**
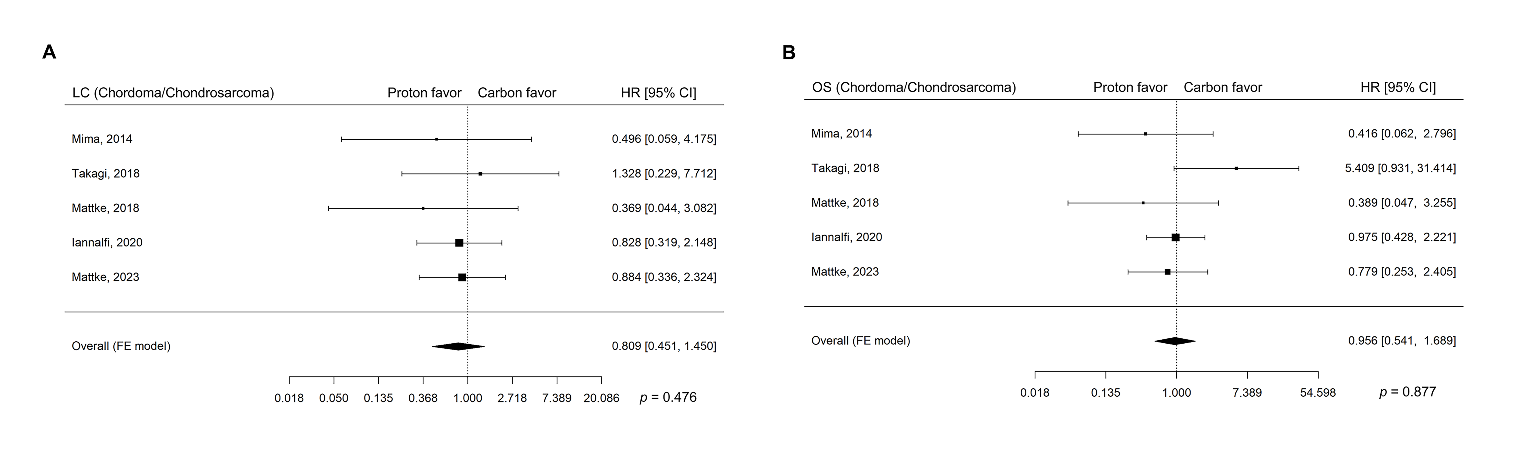
**

**Supplementary Figure S4.** Forest plots with fixed effect model of pooled analyses involving patients treated with head and neck, other sites (other than head and neck), and chordoma or chondrosarcoma regarding adverse events. AE, Adverse events; OR, Odds ratio; CI, Confidence interval; FE, Fixed effect.

**
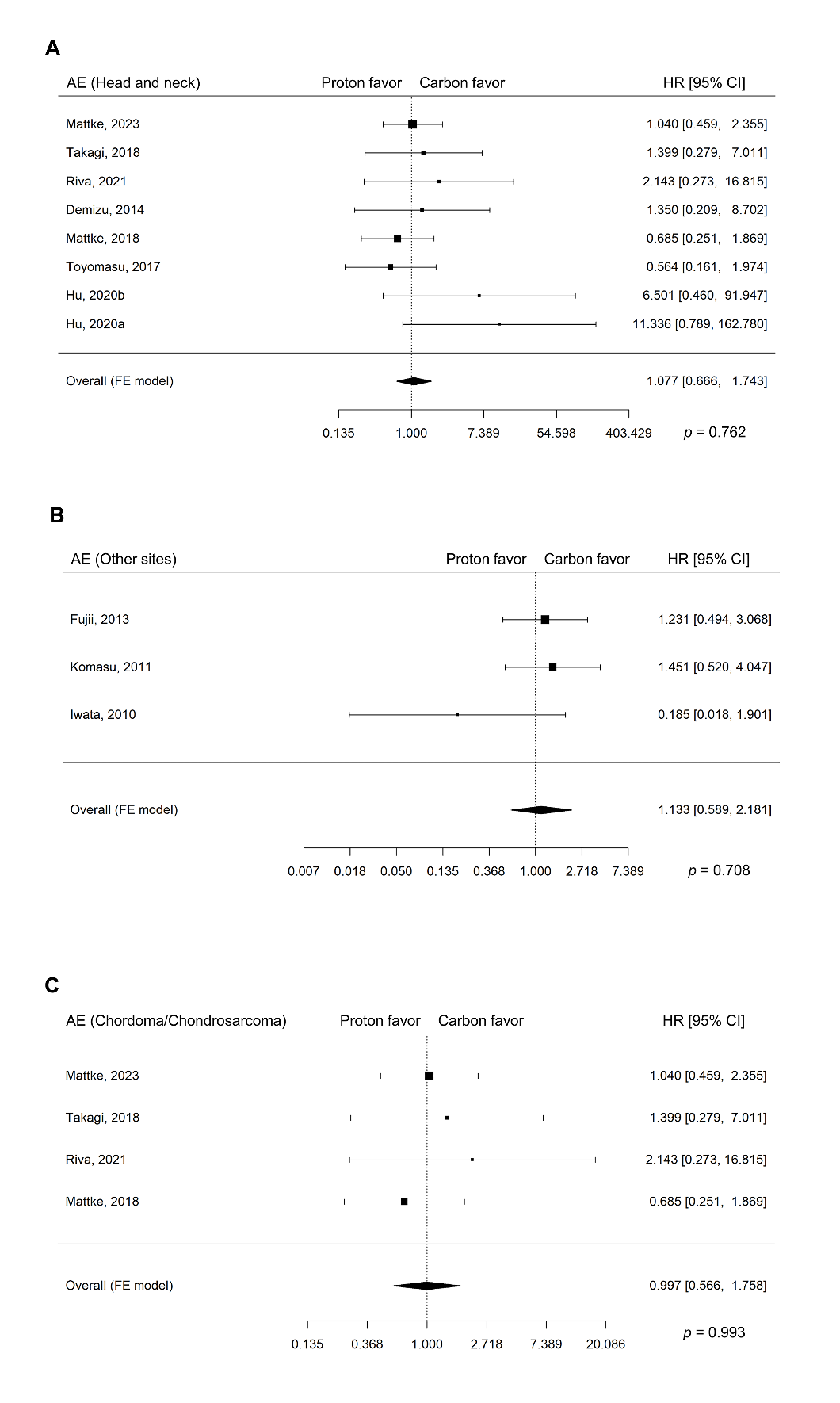
**

**Supplementary Figure S5** Forest plots with random effect model of pooled analyses regarding adverse event ≥ grade 3. AE, Adverse event; OR, Odds ratio; CI, Confidence interval; FE, Fixed effect.

**
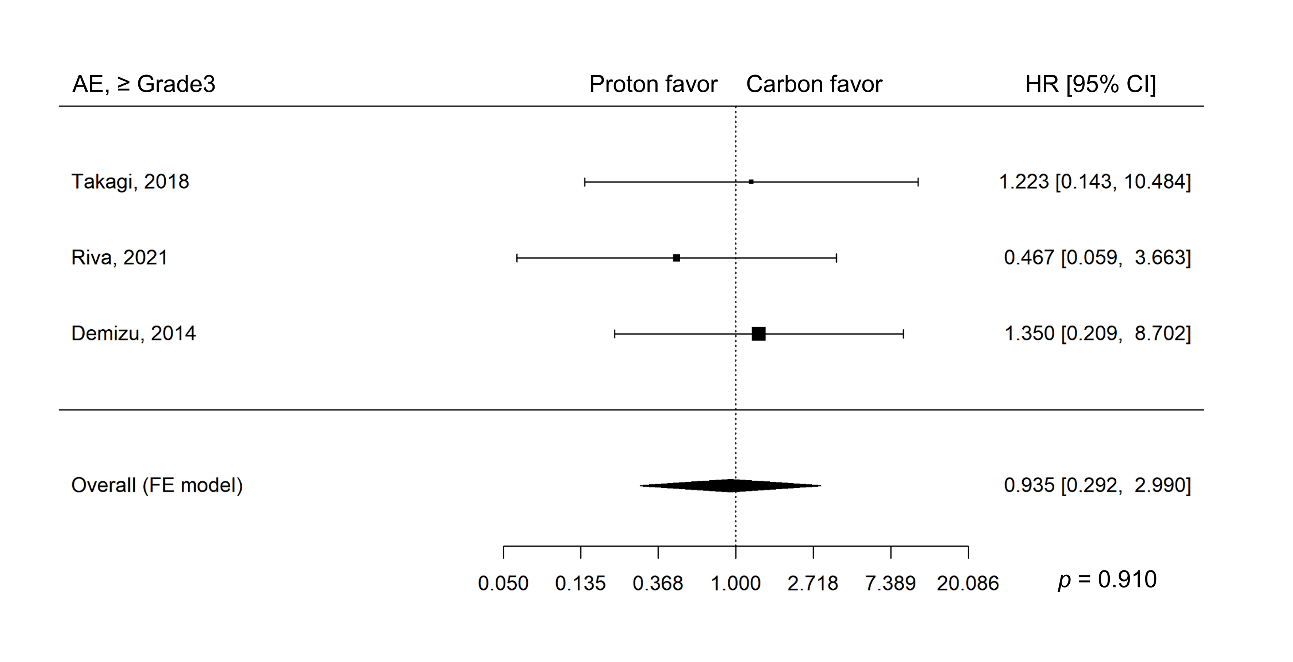
**

**Supplementary Figure S6** Meta-regression evaluating the effect of radiation dose on the occurrence of (A) local control, (B) progression-free survival, (C) overall survival, and (D) adverse events. LC, Local control; PFS, Progression-free survival; OS, Overall survival; AE, Adverse event; BED, Biologically effective dose.

**
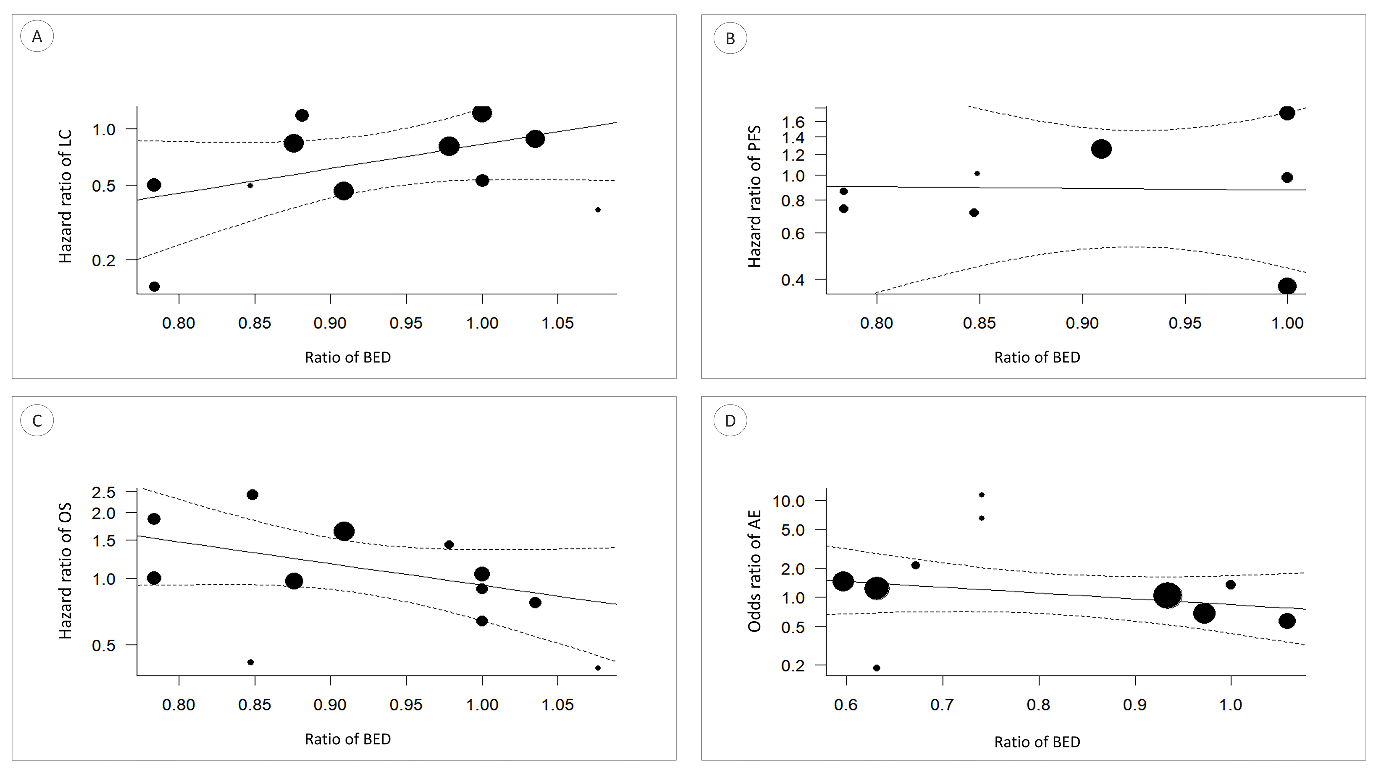
**
