# Supplementary material for: A meta‐analysis comparing efficacy and safety between proton beam therapy versus carbon ion radiotherapy
Source: Cancer Med. 2024 Feb 23;13(3):e7023. doi: 10.1002/cam4.7023 (PMC10891363; doi:10.1002/cam4.7023)
Supplement: Supplementary file 2 — Table s1. Table s2. Table s3. [file CAM4-13-e7023-s001.docx]

**Supplementary Table S1** Egger’s regression test for funnel plot asymmetry.

| **Outcome** | **t-value** | ***p*-value** |
| --- | --- | --- |
| Local control | -1.0683 | .311 |
| Progression-free survival | 0.3867 | .711 |
| Overall survival | -0.554 | .591 |
| Adverse event, any grade | 1.129 | .288 |
| Adverse event ≥ Grade3 | -0.392 | .762 |

**Supplementary Table S2** Studies included in the pooled analysis for each outcome and analysis for heterogeneity with subgroup.

| **Outcome** | **No. of**  **included study** | **No. of patients (PBT/CIRT)** | **Heterogeneity** | |
| --- | --- | --- | --- | --- |
|  |  |  | ***p*-value** | **I^2^ (%)** |
| Local control |  |  |  |  |
| Treated site: Head and neck | 6 | 549 (207/342) | .859 | 0.0 |
| Treated site: Others | 6 | 673 (375/298) | .347 | 0.5 |
| Pathology: Chordoma, Chondrosarcoma | 5 | 430 (141/289) | .900 | 0.0 |
| Progression-free survival |  |  |  |  |
| Treated site: Head and neck | 3 | 166 (84/82) | .464 | 0.0 |
| Treated site: Others | 6 | 796 (500/296) | .120 | 44.7 |
| Overall survival |  |  |  |  |
| Treated site: Head and neck | 6 | 549 (207/342) | .413 | 0.0 |
| Treated site: Others | 7 | 1066 (645/421) | .709 | 0.0 |
| Pathology: Chordoma, Chondrosarcoma | 5 | 430 (141/289) | .260 | 0.0 |
| Adverse event, any grade^*^ |  |  |  |  |
| Site: H&N, Skull base | 8 | 535 (179/356) | .412 | 0.0 |
| Site: Others | 3 | 514 (349/165) | .275 | 0.0 |
| Pathology: Chordoma | 4 | 320 (101/219) | .742 | 0.0 |

I² ≥ 50% suggests high heterogeneity across studies.

**Supplementary Table S3** Meta-regression analysis with mixed effect model.

| **Outcome** | **Covariate/ model** | **Regression coefficient** | | **t-value** | ***p*-value** |
| --- | --- | --- | --- | --- | --- |
|  |  | **Estimate** | **Standard error** |  |  |
| Local control | BED ratio | 3.0138 | 1.7913 | 1.6825 | .127 |
|  | BED ratio, Permutation | 3.0138 | 1.7913 | 1.6825 | .113 |
| Progression-free survival | BED ratio | -0.1476 | 2.5239 | -0.0585 | .955 |
|  | BED ratio, Permutation | -0.1476 | 2.5239 | -0.0585 | .975 |
| Overall survival | BED ratio | -2.2698 | 1.3955 | -1.6266 | .135 |
|  | BED ratio, Permutation | -2.2698 | 1.3955 | -1.6266 | .115 |
| Adverse event, any grade | BED ratio | -1.3756 | 1.1842 | -1.1616 | .279 |
|  | BED ratio, Permutation | -1.3756 | 1.1842 | -1.1616 | .143 |

Abbreviation: BED, Biologically effective dose.

The BED ratio is calculated by dividing the BED of proton beam therapy by the BED of carbon ion radiotherapy.
